# The Influence of Selected Food Safety Practices of Consumers on Food Waste Due to Its Spoilage

**DOI:** 10.3390/ijerph19138144

**Published:** 2022-07-02

**Authors:** Marzena Tomaszewska, Beata Bilska, Danuta Kołożyn-Krajewska

**Affiliations:** Department of Food Gastronomy and Food Hygiene, Institute of Human Nutrition Sciences, Warsaw University of Life Sciences-SGGW, 159C Nowoursynowska St., 02-776 Warsaw, Poland; beata_bilska@sggw.edu.pl (B.B.); danuta_kolozyn_krajewska@sggw.edu.pl (D.K.-K.)

**Keywords:** households, hygiene, prevention of food waste, food spoilage, food storage, food security

## Abstract

Food waste in households is a consequence of the accumulation of improper practices employed by consumers when dealing with food. The survey estimated the impact of practices of Polish respondents, in the context of selected food safety and hygiene issues, on throwing away food due to spoilage. The survey was conducted in 2019, in a random quota-based, nationwide sample of 1115 respondents 18 years old and older. Synthetic indicators (SI) were created to assess the knowledge and practices of Polish adult respondents concerning selected areas of food management and the frequency of throwing food away. Most food products were not thrown away at all or were thrown away occasionally. Regression analysis revealed that the frequency of throwing food away was to the greatest extent related to food spoilage (β = 0.223). Among the five areas of Polish respondents’ practices covered by the analysis, the most conducive to wasting food due to spoilage were improper proceedings with food after bringing it home (β = 0.135; *p* = 0.000), a failure to ensure proper food storage conditions (β = 0.066; *p* = 0.030), or inappropriate proceedings with uneaten meals, excluding the food plate (β = 0.066; *p* = 0.029). To reduce food waste in Polish households, drawing the attention of consumers to the conditions of food storage at home seems appropriate. It is also vital to convince them to use freezing of uneaten food as an effective method of extending the life of food products.

## 1. Introduction

Food is a basic necessity for people as it provides them with survival, proper development and health. Meanwhile, access to food is still partially or significantly limited in many regions of the world, which threatens the well-being of societies. It was estimated in 2020 that from 720 to 811 million people worldwide experienced hunger, which is as much as 161 million people more than the year before considering the upper end of this range [1]. The struggle with numerous waves of the pandemic in 2021 and early 2022, as well as the military conflict in the eastern part of Europe (from 24 February 2022, the beginning of the Russian Federation’s invasion of Ukraine), will probably deepen inequalities in access to food. Therefore, food security, which should be achieved at individual, household, national, regional and global levels will still remain unguaranteed [2]. In the face of food insecurity, attention is drawn to the scale of food loss and waste (FLW), which relate to each link of the food chain. In the 2030 Agenda, adopted under the aegis of the United Nations (UN), food waste is primarily reflected in objective 12 concerning sustainable consumption and production (in one of the assignments it was assumed that the global amount of food wasted per person in retail sales and consumption should be halved by 2030) and indirectly in objective two, which assumes the elimination of hunger, achieving food security and better nutrition as well as the promotion of sustainable agriculture. 

### 1.1. The Scale of Food Loss and Waste

According to the 2011 report of the Food and Agriculture Organization of the United Nations [3], one third of food suitable for eating by humans was wasted every year in the first decade of the 21st century. Including the global mass of food production, it was about 1.3 billion tons a year. The report “A preparatory study on food waste across the EU 27” [4], developed mainly on the basis of Eurostat data for 2006 and other national sources considering expert assessment, shows that, in 2006, 27 countries of the EU wasted 89.3 million tons of food. Searchinger et al., [5] pointed out that Europe is responsible for 22% of food waste on a global scale. The only data found in Poland, concerning the scale of food loss and waste, came from the Eurostat database and were from 2006. According to these estimates, Poland wasted almost nine million tons of food per year. However, a recent survey has revealed that the estimates concerning Poland were considerably overstated. Łaba et al., (2020) have proven that over 4.8 million tons of food are wasted in Poland every year. The results obtained have additionally pointed out that the greatest amount of food (60%) is wasted by consumers in households [6]. The authors conducting their research in other countries, especially those with a high income [7,8], have also proven that households, as a link of the food chain, are the most responsible for food waste [9,10,11,12,13,14,15,16,17,18,19]. It was estimated that in developed countries about 198.9 kg of food is wasted per person every year [20]. The amount of food wasted in households in Finland, Denmark, Norway and Sweden constituted 30%, 23%, 20% and 10-20% of purchased food, respectively [21]. Other data show that about half of the food wasted could have been avoided, e.g., in Canada 52% [22] or in Hungary 48.7% [23]. Therefore, understanding the mechanisms concerning food waste in households is a matter of utmost importance.

### 1.2. The Multidimensional Problem of Food Loss and Waste

The problem of food loss and waste is becoming increasingly recognized in the international forum, especially in the context of the foreseen population growth. International organizations have calculated that to feed the expected world population of over 9500 million in 2050 [24], the global food system would have to increase food accessibility by about 70% [25,26]. The issue of food waste is important not only because of negative social effects [9], but also economic [7,27,28,29] or environmental ones [30,31]. Based on a preliminary assessment of the full costs of food wastage on a global scale, it was found that, in addition to the USD one trillion of economic costs per year, environmental costs reach around USD 700 billion and social costs around USD 900 billion [28]. The costs associated with food waste for the EU-28 in 2012 were estimated at around 143 billion euros. Two-thirds of the cost was associated with food waste from households (around 98 billion euros) [7]. Considerable amounts of food waste represent considerable amounts of unnecessary global food production, resulting in high amounts of unnecessary global environmental impacts, like unnecessary greenhouse gas emissions, water consumption and land use [32]. The quantities of food waste in Europe correspond to approximately 186 million tons of CO_2_ equivalents per year [33]. It was estimated that about 23–24% of water is used to produce wasted food [34]. The importance of this matter is reflected in a constantly growing number of publications on this subject [35,36].

### 1.3. The Most Wasted Food Products

The most frequently wasted food products, in the case of consumers, are bread, fresh fruit, vegetables, smoked meats and dairy products. The mentioned groups of products are indicated by authors conducting research in different countries, sometimes in a different order [15,37,38]. These groups of food products belong to so-called perishable food. Perishable food refers to a category of commodities that are subject to quality damage during their manufacturing stage, storage, shipping, or handling. These kind of food products are temperature-sensitive and, therefore, are unusually susceptible to spoilage during processing, packaging, transportation, and handling [39,40]. The rate at which food products spoil can be influenced by many factors, e.g., the failure to comply with process conditions and individual operations, as well as hygiene standards, during processing. For example, abusive storage conditions result in the microbial spoilage of foods, hence these products are not consumed [41]. As emphasized by Heng and House [42], fresh fruit and vegetables are highly perishable and more likely to become inedible and dumped. The appearance of fruit and vegetables, their firmness or color change over time, especially when they are stored in inappropriate conditions. In the research of Heng and House [42], about 60% of customers reported throwing away fruit and vegetables since ‘they got spoiled more quickly than expected’. The products visually diverging from commonly accepted standards, for example those deformed, distorted or inadequately colored are wasted by respondents [43,44]. As emphasized by Heng and House [42] customers are guided by the look of food to determine whether it is spoiled or not. 

### 1.4. Factors Influencing Food Waste

Several factors have an influence on the amount of food wasted by customers. They are socio-demographic, cultural and environmental factors. The prevention of food waste among customers is not only a multi-dimensional matter but also a multi-phase one. Stimulating customers to reduce food waste demands the profound exploration of behavioral factors as well as obstacles constraining the alternation of unfavorable practices [45]. In many studies, great emphasis is placed on understanding the motivations and practices of consumers in terms of food waste. The practices are usually analyzed in areas such as planning purchases (the observation of stored food and the preparation of a shopping list), the proceedings of shopping (avoiding shopping on impulse), the storage of some fruit and vegetables in a fridge, the preparation of adequate amounts of meal and leftover usage [42,45,46]. The conditions in which food is purchased, processed and stored also influence the shelf life of food products. Therefore, non-compliance of customers to the basic practices concerning food safety and hygiene cannot only cause food poisoning [47,48,49], but may also lead to food spoilage and, at the same time, decrease the level of food security [50]. Heng and House [42] emphasized the fact that customers do not waste food intentionally. According to van Gaffen et al. [51], food waste by customers results from the accumulation of different household habits concerning food and there is no single practice on which one should concentrate to limit wastage. 

### 1.5. Objectives

This work concentrates on dependencies between good safety/hygiene practices while purchasing food and proceeding with it at home in the context of food waste due to spoilage. Therefore, it presents an alternative approach to the problem of food waste by customers in households since food spoilage is one of the most often reported reasons for throwing food away by customers [15,52,53,54,55]. 

**Figure 1:**
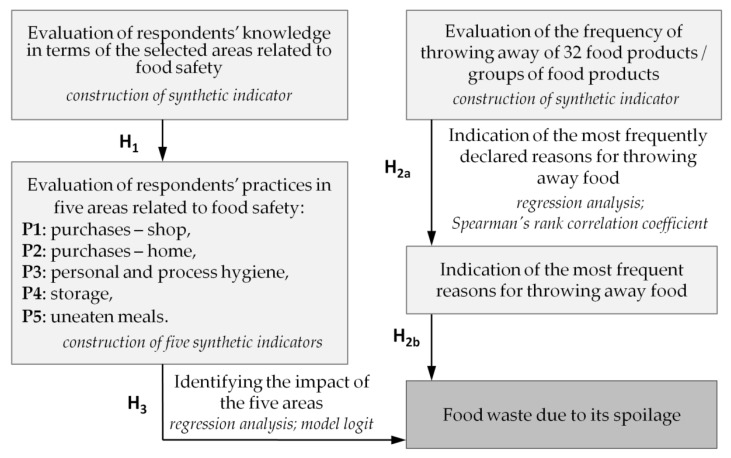
The conceptual research model adopted in the work.

The aim of this work was to determine whether the selected areas of consumer practices, in terms of food safety/hygiene, result in wasting food due to spoilage. This work constitutes an extension to the previous research conducted in Poland [53,56,57] explaining consumer practices related to food waste as well as the impact of socio-demographic characteristics. Figure 1 shows the conceptual research model. The following research hypotheses were formulated in this work:

**Hypothesis** **1** **(H1).** *The level of knowledge of respondents in terms of food safety finds its reflection in their proceedings with food while shopping and after bringing it home.*

**Hypothesis** **2a** **(H2a).** *Polish consumers hardly ever throw away food.*

**Hypothesis** **2b** **(H2b).** *If they throw away some food it is mainly due to spoilage.*

**Hypothesis** **3** **(H3).** *Complying with the basic rules of food safety, while shopping for food, storing it, preparing meals and dealing with uneaten items, equally decreases food waste in households due to spoilage.*

## 2. Materials and Methods

### 2.1. Study Design and Participants

The data used in this research were collected in Poland, in 2019, as a part of the project entitled “Developing a System for Monitoring Wasted Food and an Effective Program to Rationalize Losses and Reduce Food Wastage” (2018–2021) [53].

The survey was conducted with the employment of a random quota-based, nationwide sample of 1115 respondents 18 years and over. The minimum sample size (*N_min_*) was calculated in accordance with Equation (1) [58].

(1) $$N_{min}=\frac{Np(\alpha^{2}\cdot f\left(1-f\right))}{Np\cdot e^{2}+\alpha^{2}\cdot f\left(1-f\right)}$$

where: *Np* means the size of the sampled population; α—confidence level; f—fraction size, e—assumed maximum error.

**Figure 2:**
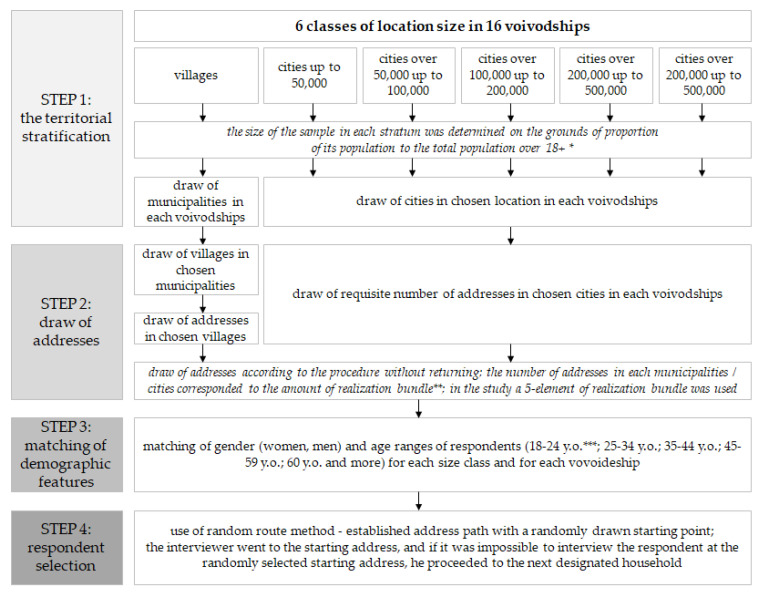
The phases of the sample selection procedure. * demographic data being the basis of territorial stratification come from the Central Statistical Office [63]; ** realization bundle—includes a group of spatially clustered addresses—most often selected in one town [64]; *** y.o.—years old.

Participant recruitment and data collection were carried out by a professional market research agency, respecting the ESOMAR (the European Society for Opinion and Marketing Research) code [59]. The sample fulfilled the condition of representativeness of the general population for Poles aged 18 years and over in terms of gender, age, and the place of residence of the respondents. The sample was selected from the TERYT address survey (National Register of the Official Territorial Division of the Country) kept by the Central Statistical Office [60]. The TERYT database contains the addresses of buildings and flats in the structure of statistical regions. This database is used to draw a sample in most projects implemented on address samples of Polish residents [61,62]. In the first phase of the sample selection procedure, the territorial stratification of the population was carried out, taking into consideration voivodeships (16 voivodships) and size classes of locations (six classes). The next phases of the sample selection procedure are shown in Figure 2. This kind of sample selection procedure ensures that research is representative. The structure of the sample in terms of gender, age, place of residence, or voivodeship does not differ significantly from the entire Polish population.

Table 1 presents the socio-demographic characteristics of the respondents. The number of men and women participating in the survey was comparable. The smallest group, in terms of age, was the group with the youngest respondents, i.e., aged 18–24. The biggest groups, in terms of age, were respondents aged 45–59, as well as 60+. Among respondents, most of the people were those of secondary education. Even fewer than every fifth respondent reported to be highly educated. The greatest number of respondents came from the countryside.

### 2.2. Data Collection

The survey was conducted at the homes of respondents by the use of the Computer Assisted Personal Interview (CAPI) [65]. The interview was conducted by trained pollsters. The pollsters were directly subordinate to regional coordinators, which were under the central unit of the professional market research agency. The pollsters were trained on the methodological assumptions of the study and on how to use the electronic version of the research tool. During the completion of the questionnaire, the interviewers could resolve any doubts that consumers might have [48]. Permanent contact of pollsters with regional coordinators was ensured. The results of the work of pollsters underwent the following control. The control was carried out by the use of CATI (Computer Assisted Telephone Interview). The percentage of checked interviews constituted 10% (*n* = 115). During this control discrepancies were observed in the case of four interviews (i.e., a lack of a pollster’s ID badge, suggesting answers by a pollster or interviewing respondents beyond their place of residence). These data were deleted from the database and other respondents were interviewed. 

### 2.3. Questionnaire

**Figure 3:**
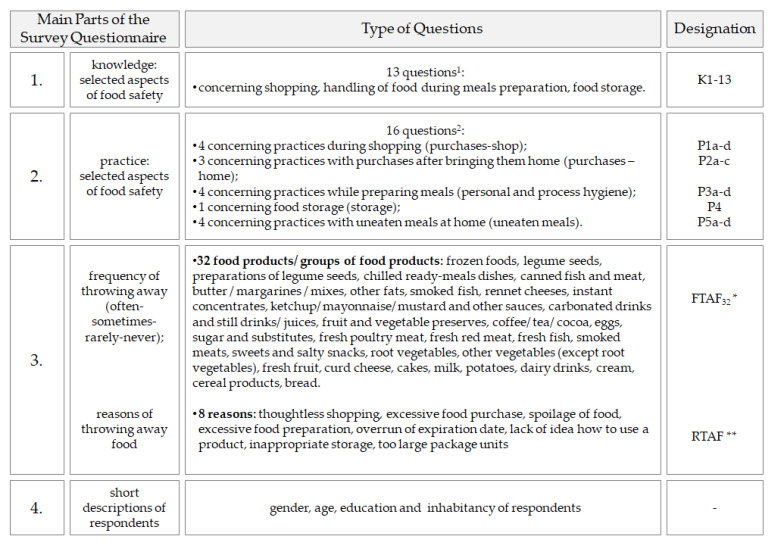
Division of the questionnaire into four parts with the adopted designation.^1^ Appendix A—Table A1; ^2^ Appendix A—Table A2 and Table A3; * FTAF_32_—Frequency of Throwing Away Food; ** RTAF—Reasons of Throwing Away Food.

The questionnaire consisted of four parts, which concerned: (1) knowledge, (2) customer practices in terms of selected aspects of food safety, as well as (3) the frequency of throwing away food and the reasons for it, and (4) short descriptions enabling the socio-demographic characteristics of the respondent population (Figure 3). The first and second part of the designed questionnaire were prepared on the basis of Codex Alimentarius (CA) general principles of food hygiene [66]. The third part of the survey was designed based on literature and previous research [67,68].

### 2.4. Statistic Methods Applied

#### 2.4.1. The Construction of Synthetic Indicators (SI)

In order to verify hypothesis 1–3, synthetic indicators were constructed to collectively describe the knowledge (K1–13) of respondents as well as selected areas of their practices (P1–P5) in terms of food safety and frequency of throwing away 32 groups of food products (FTAF_32_). The synthetic indicator is a numerical measure reflecting the situations of an objective state of affairs made up by many meant to be integrated into a single comprehensive value [69]. The construction of synthetic indicators was conducted with the use of an Excel spreadsheet.

While constructing the synthetic indicators, firstly the variables (features) concerning the knowledge of respondents and selected areas of their practices were grouped alongside estimating their reversal pointing out the right answers. The synthetic indicator concerning the knowledge of respondents was constructed on the basis of 13 statements (K1–13). In the part concerning dealing with food in terms of food safety, five synthetic indicators were constructed: (Figure 3), i.e.,:

1. practices during shopping (P1a–d) (adopted name: purchases-shop),
2. practices while dealing with purchases after bringing them home (P2a–c) (adopted name: purchases-home),
3. practices while preparing meals (P3a–d) (adopted name: personal and process hygiene),
4. practices while preparing right storage conditions (P4) (adopted name: storage),
5. practices while dealing with uneaten meals at home (P5a–d) (adopted name: uneaten meals).

As for the part concerning food wasting, the synthetic indicator was built on the basis of the declared frequency of throwing away of 32 different food products (FTAF_32_). 

Since the answers to the questions were not based on the same scale, it was necessary to carry out a standardization process to obtain comparable values. The standardization process demanded the calculation of weight for every variable and afterwards their ranking. The weight for each question was calculated in accordance with Equation (2), where *f_i_* for *i* = 1, …, *k* denotes the frequency of answers considered correct to the *i*-th question among the respondents.

(2) $$V^{i}=\frac{\left(1-f_{i}\right)}{\sum_{i=1}^{k}\left(1-f_{i}\right)},\;i=1,\ldots ,\;k$$

As for the answers ranking, the function “POSITION.AVG” available in the Excel spreadsheet was applied. In case of the part concerning knowledge of respondents, while ranking questions K4, K5, K8, the order of the scale was reversed. In the case of the part concerning practices of respondents, the order of the scale was reversed while ranking questions P2c, P3a, P5a, P5b. Weights and ranks calculated for various statements/ questions are presented in Table A1 and Table A2 (Appendix A).

Constructed on the basis of ranks of the answers together with their weights, the synthetic indicator calculated for each respondent was presented in the form of the sum of the weighted ranks divided by the number of respondents (*n* = 1115), so that the values were in the range of (0; 1). A value closer to one indicates a greater percentage of correct answers.

While interpreting obtained synthetic indicators (SI) for various areas of knowledge and practices, a grading scale was adopted, and the SI range from 0.9 to 1.0 meant a very good grade. However, while discussing the synthetic indicators calculated for the frequency of throwing away 32 different food products, the rating scale was reversed, i.e., the SI range from 0.9 to 1.0 accounted for the unsatisfactory assessment of the frequency of wasting food (Table 2).

#### 2.4.2. Regression Analysis

Regression analysis was used to explain the influence of practices (from five selected areas) of Polish adult respondents on food waste due to its spoilage. The regression analysis was also used to check which reason for throwing away food, reported by respondents, has the greatest influence on the reported frequency of wasting food. 

Moreover, to check the correlation between the most often wasted food products (condition: answers ‘often’ and ‘sometimes’ at a level above 10% of indications) and most often reported reasons for throwing away food, Spearman coefficients of correlation were calculated. 

Regression analysis and Spearman correlation were performed using Statistica 12.1 software (StatSoft, Cracow, Poland). This verification was performed at a significance level of α = 0.05.

#### 2.4.3. Model Logit

While analyzing the influence of practices of respondents dealing with food in five different areas (explanatory variables) on waste food due to its spoilage (the dependent variable), a logit model (a qualitative model) was used [70], in which the dependent variable (Y) was “throwing away food due to its spoilage”. Variable Y in the logit model took the following values:

Y = 1 − reporting spoilage of food as the reason for throwing it away,

Y = 0 − no report of food spoilage as the reason for throwing it away.

The results of the logit model estimation were interpreted based on: (a) the log odds ratio (LOR), (b) the odds ratio (OR) for each independent variable, through which the change in the odds of occurrence of the selected value is expressed (Y = 1) when the independent variable grows by 1 unit (ceteris paribus).

The quality of matching the model of the variable “throwing away food due to its spoilage” was evaluated on the basis of the Maddal [71] determination coefficient (R^2^). The values of this coefficient fall within the range (0.1), and the higher the value, the better the matching of the model.

## 3. Results

### 3.1. Rating of Knowledge and Practices of Respondents in the Context of Selected Issues Concerning Food Safety Based on Calculated Synthetic Indicators (SI) 

On the basis of the mean value of SI, it was stated that the knowledge of Polish respondents (K1–13) and all five areas of practices employed while dealing with food (P1, P2, P3, P4, P5) can be rated at a satisfactory level (Table 3). The highest mean value of SI, indicating the highest percentage of proper answers, was observed in the case of questions concerning practices of respondents while dealing with purchases at home (P2: 0.681).

Taking the distribution of synthetic indicators into consideration, calculated individually for each respondent, it was observed that none of the people participating in the survey presented a very good level of knowledge concerning issues raised in the questionnaire. What is more, the knowledge of only 6% of adult Polish respondents was rated at a good level. More than a half of respondents answered at an unsatisfactory level (SI under 0.5: 54.08%). The most problematic for respondents was to come up with the correct answer in the case of statements: K7–concerning the perception of storage of leftovers looking “fine” and/or those having a typical smell as those which are safe and edible (20.3% correct answers), K13 concerning the depiction of the best place in the fridge to store raw minced meat (29.3% correct answers) and K12 concerning the temperature at which poultry should be stored in a refrigerated store display (37.0% correct answers) (Table A1). Moreover, about three quarters of the respondents knew that by washing fruit and vegetables, the number of microorganisms present on their surface can be reduced (K4), and that the cutting board, on which raw meat was cut, should be washed immediately by hand or mechanically (K8) and that defrosted products, for example, unused meat must not be frozen again.

Taking the distribution of the synthetic indicators into consideration, calculated individually for each respondent in the case of five distinct areas of practices employed while dealing with food, it was observed that more than half of Polish adult respondents got an unsatisfactory grade (SI from 0.0 to < 0.5) in the case of practices employed while storing food products (P4: 57.13%), dealing with uneaten meals (P5: 52.29%) and purchasing food in a store (P1: 51.93%). Moreover, a great percentage of respondents (nearly 50%) gave unsatisfactory answers in the case of personal hygiene and the hygiene of implemented processes (P3: 49.96%) (Table 3). The highest percentage of respondents received a positive grade (an SI higher than 0.5) in the case of dealing with purchases brought home since one out of ten respondents participating in this survey got a very good grade and four out of ten received a good grade. As the data presented in Table A3 show, respondents most often reported inappropriate conditions of storage for such products as UHT milk, lettuce, opened bottles of juice, tomatoes or carrots. In the case of practices employed while dealing with uneaten meals (P5), only a little more than 15% of respondents reported freezing uneaten food, and almost half of them (49.4%) reported placing it in a fridge after cooling down at room temperature. Additionally, 46% of Polish adult respondents reported that they left uneaten meals in pots on a cooker and/or in an oven until eating them (Table A2). The inappropriate practices of Polish customers in the process of purchasing food (P1) were also observed. Even though, for almost three quarters of respondents, the storage conditions advised by the producer on the label are ‘definitely’ or ‘rather’ important, only two out of ten people pay attention to the temperature of the fridge/refrigerating counter/freezer at which the products are stored in a shop or use thermo-insulating bags while shopping for frozen food. It was also observed that two thirds of customers do not pay great attention to placing the products requiring the preservation of the cold chain (non-shelf stable products) in a basket at the end of shopping.

### 3.2. Reported Frequency of Throwing Away Food Products and Its Reasons

The highest mean value of the synthetic indicator (SI from 0.9 to 1.0), pointing to unsatisfactory i.e., fairly regular, frequency of wasting food products (FTAF_32_), was obtained only for less than 2% of respondents participating in this survey (Table 4). The frequency of throwing away 32 different food products, for the vast majority of respondents, was graded at a very good or good level (84.66% in total), which proves that most of the food products included in the analysis were not thrown away at all or were thrown away occasionally.

**Figure 4:**
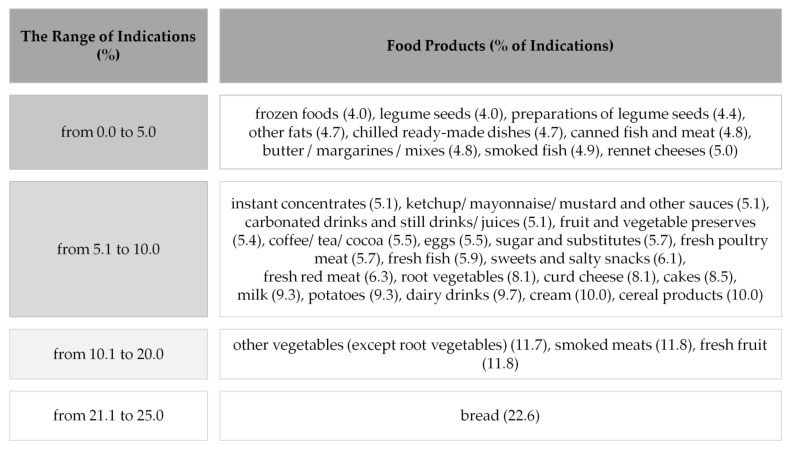
Food products wasted by Polish respondents (*n* = 1115) with frequency ‘often/sometimes’.

The greatest number of respondents reported the frequency of throwing away bread using the words ‘often’ and ‘sometimes’ (Figure 4). The smaller number of Polish customers reported the waste of fresh fruit, vegetables and cold meats. Whereas the smallest percentage of respondents used the words ‘often/sometimes’ while reporting throwing away frozen foods, legume seeds (fresh or preserved) and chilled ready-made dishes.

The most frequently reported reason for throwing away food was its spoilage (over 50% of answers). The analysis of regression additionally revealed that the reported frequency of throwing away food (with the use of words ‘often’ and ‘sometimes’) was to the greatest extent related to food spoilage (β = 0.223) and, next, to thoughtless shopping (β = 0.202) (Table 5). 

On the basis of the obtained results, it was stated that the constructed model explains 23.4% (R^2^ = 0.234; *p* = 0.000) of fluctuation of the synthetic variable estimating the frequency of throwing away food. 

Calculated coefficient correlations of Spearman showed that Polish respondents, reporting the increasing frequency of throwing away food products from four main groups i.e., bread, fresh fruit, cold meats, vegetables (apart from root ones) (Figure 4), more often pointed to spoilage of food as a reason for throwing it away—the highest correlations of the Spearman rank (r) (Table 6). Although it was a weak correlation, it was a significant one (0.2 < r > 0.4). Therefore, hypothesis 2b, concerning throwing away food by respondents mostly due to its spoilage, was confirmed. 

### 3.3. The Specification of the Influence of Respondents’ Practices, in Terms of Selected Issues Concerning Food Safety, on Food Waste Due to Its Spoilage

The results of regression analysis showed that reported practices, while dealing with purchases at home (P1), were significantly related to throwing away food due to its spoilage (β = 0.135; *p* = 0.000). Also, storage (P4) and dealing with uneaten meals (P5) were, to a lesser extent, significantly related to waste food due to spoilage (P4: β = 0.066; *p* = 0.030; P5: β = 0.066; *p* = 0.029). However, it turned out that the reported practices performed by respondents while shopping (P1) and those concerning personal and process hygiene (P3) did not have a significant influence on throwing food away due to its spoilage (Table 7).

Regression analysis was a preliminary assessment of the influence of different areas of practices (P1–P5) on throwing food away due to its spoilage (the dependent variable). Since the dependent variable is on a qualitative scale, the logit model was applied as a tool, which confirmed the results of regression analysis (Table 8). The positive direction indicates that P2, P4 and P5 are significantly conducive to (*p* < 0.05) throwing away food due to its spoilage. Practices performed while purchasing food in the store (P1) are not conducive to the discussed phenomenon. The values of the odds ratios (OR) additionally indicate that, whereas in the case of models P2 and P5 the increase in the chance of throwing food away due to spoilage is minimal (for P2-0.016%; for P5 approx. 0.008%), storage (P4) increases this chance by almost 2.5 times (2.44). Therefore, hypothesis three has not been positively verified, as the diagnosed areas of practices (P1–P5) do not have a comparable influence on throwing away food due to spoilage.

Table 9 presents the number of hits of the predicted values 0 and 1 in comparison to actual values. There were more than 65% of recognized indications Y = 1 correctly classified in the logit model.

The Maddal enumeration correlation coefficient, as a measure of the model’s matchmaking, was almost 0.59 (R^2^ = 0.588340807). It proves a fairly good match of the model describing the alternation of the value of the variable “throwing away food due to spoilage”.

## 4. Discussion

Based on the synthetic indicators constructed for this survey, it was stated that a significant number of Polish customers represented an insufficient level of knowledge and reported inappropriate dealing with food in terms of its safety and hygiene, which allowed us to positively verify H1. Also, customers from other countries, to a different extent though, had only partial knowledge and employed inappropriate practices in the above-mentioned aspect [72,73,74]. A sizable percentage of adult customers surveyed obtained an unsatisfactory grade for food storage, dealing with uneaten meals at home, or paying attention to the preservation of the cold chain when purchasing frozen foods. In the case of every mentioned area of practices (respectively: P4, P5, P1), according to the reports of some customers, the food products purchased by them were not guaranteed the proper temperature conditions. Meanwhile, this parameter constitutes an effective tool limiting the growth of microorganisms in food. Nonadherence to the recommended temperature values is the main cause of the multiplication of microbial cells and, in consequence, numerous threats, including food poisoning [75], or more rapid spoilage of food [76]. Many studies focused on the inappropriate practices of customers in the case of controlling temperature while preparing and storing food at home [77,78,79,80]. According to Terpstra et al., [81] customers do not always store vegetables in appropriate conditions or the temperature in their refrigerators is too high. Moreover, Polish customers often reported inappropriate storage conditions of vegetables, such as lettuce, carrots, but also other products, such as UHT milk or opened bottles of juice.

However, it was found that Polish respondents do not report throwing away food too often. The synthetic indicator, constructed on the basis of the reported frequency of throwing away food from 32 different food groups, allowed us to positively verify hypothesis H2a. Moreover, consumers in other countries like the United States, Canada, the United Kingdom or France, when asked about the frequency of throwing away fruit and vegetables, most often used the word ‘rarely’ [42]. Italian customers also reported wasting a small amount of food [82]. As emphasized by Jribi et al., [83], respondents tend to significantly under-report the level of food waste as opposed to over-reporting their efforts to reduce it. Nevertheless, even the occasional throwing away of some food products causes Polish customers to waste almost three million tons of food every year [6]. The most frequently wasted product, in the case of Polish customers, turned out to be bread. What is more, in other surveys conducted in Poland [84,85], it was found that bread is the food product most often dumped by customers. The frequent throwing away of bread was also reported in other European countries (Spain, Germany and the Netherlands) [51] but also in Africa (Tunisia) [83]. It should be emphasized that, in the author’s own research, respondents reported appropriate conditions of bread storage (96% of appropriate responses). Therefore, it should be assumed that, in the case of this product, the main reason for throwing away food was a mismatch between the purchased amount of food and its real consumption. The limited shelf life of bread [86,87] and the fact that customers perceive its freshness as the most important feature of its quality [88] make bread one of the most often dumped food products along with fresh fruits and vegetables or cold meats [15,37,38].

Not only the author’s own research, but also the research of other authors [8,52,54] show that customers dump food mainly due to its spoilage. The process of food spoilage makes the product impossible to be eaten by a customer [89]. Thus, it is one of the factors contributing to a lack of food security in many regions of the world [50]. Food spoilage results from many external factors as well as from the characteristics of a product (pH, water activity, percentage of nutritious constituents), its initial microbiological load, way of packaging and storage conditions [50,89]. The statistical tools applied here allowed to show that, along with the increasing frequency of throwing away bread, fresh fruits, fresh vegetables and cold meats reported by consumers, the most often reported reason for food waste was its spoilage. Therefore, the second part of hypothesis two was also positively verified. Moreover, it was also proven that the frequency of throwing away vegetables (rootless) and fresh fruit was more strongly connected with food spoilage than in the case of other products. Attention should be paid to fruit and vegetables, as those were among the food products most commonly dumped due to their spoilage, in the context of the COVID-19 pandemic. The research showed that eating habits have changed, for example, customers prepare meals at home more often [83,90] and they do their best to include more fruit and vegetables in their everyday diet [91]. As proven in the research of Everitt et al., [22] this change caused customers to dump vast amounts of fruit and vegetables.

Appropriate statistical tools were applied to search for the answer to the question concerning the influence of respecting the basic rules of food hygiene and safety while shopping, storing food at home, preparing meals and dealing with uneaten dishes, on reported food waste due to its spoilage (hypothesis three). It turned out that the process of purchasing food products themself (area P1), i.e., the order of placing perishable or frozen foods in shopping baskets, paying attention to the conditions in which they are stored in a shop, does not matter in the context of its throwing away due to its spoilage. The purchasing process matters in the context of food spoilage but in terms of its planning (e.g., preparation of a shopping list), or its realization itself (purchasing too much food in relation to its real consumption) [12,92]. As emphasized by van Geffen et al., [45] food is often dumped directly after shopping. However, it most often happens at further stages of managing food at home. Also, the personal hygiene of people preparing meals and the hygiene of applied processes like defrosting, do not matter considerably in the case of food waste due to its spoilage (area P3). 

It turned out, that in terms of proper hygiene practices, the risk of spoilage and food waste is mostly connected with inappropriate storage conditions (area P4). This outcome confirmed the observations of other authors who pointed out the inappropriate storage conditions of food products (excessive time and inappropriate temperature) as one of the key determinants of food waste by consumers [83,93]. The mismatch between storage conditions and kinds of stored food results in the faster pace of food spoilage, in more or less apparent changes in its appearance, taste and smell. Meanwhile, as emphasized before, the food products visually diverging from commonly accepted norms [43,44] or those alarming in terms of their safety [94] are rejected by the customer, which subsequently escalates the level of food waste. Heng and House [42] observed that French and American respondents, who knew how to store and freeze food, dumped it less frequently. Apart from complying with the appropriate parameters of storage another key factor is the arrangement of food products in a fridge. The random and unsystematic arrangement of food in its storage space causes it to be easily “lost” and the problem of exceeding the use-by date determined by a producer emerges [93]. Polish consumers also reported that they do not eat perishable products once the use-by date is exceeded [95].

The necessity of drawing greater attention to appropriate food storage conditions, in the context of reducing food waste in households, was highlighted during the Dutch campaign organized as a part of the project named United Against Food Waste. Consumers were given a ‘Yes/No refrigerator sticker’ which was to help them decide which products should be stored in appropriate cooling conditions and which do not require any specific storage conditions. Expanding the knowledge of consumers concerning appropriate food storage conditions as well as the other subjects discussed during this campaign, including reading expiration dates, brought expected effects. Researchers from the Netherlands Nutrition Center found that average annual household food waste shrank by 7 kg between 2016 and 2019 [96].

Apart from the storage conditions, throwing away food due to its spoilage was also related to practices of respondents while dealing with prepared but uneaten meals (area P5). The small percentage of respondents reported freezing excess prepared food. Meanwhile, the appliance of the appropriate method of prolonging the usefulness of food plays an important part in reducing food waste caused by consumers [97]. Martindale [98] proved that using frozen food in households resulted in a reduction in the weight of food waste. Of course, using frozen food requires a customer’s greater attention in terms of the proper process of its defrosting. Even though, Polish consumers are generally aware of the fact that providing cooling temperatures during different processes, for example the storage of perishable food, slows down the growth and development of microbes [49]. In the survey they most often reported defrosting food by leaving it at an ambient temperature. Of course, in the scope of analysis of the received outcome (area P3), it seems that an inappropriate method of defrosting is much more important in the context of foodborne outbreaks rather than reported food waste due to its spoilage. 

## 5. Conclusions

The level of hygienic knowledge in the case of a considerable part of Polish respondents is insufficient and their practices in chosen areas of dealing with food is unsatisfactory. The synthetic indicators calculated showed that when providing food products, the appropriate storage conditions or appropriate dealing with uneaten meals are especially problematic for the respondents. According to their report, Polish respondents rarely dump food and if they do it, they are most often products like bread, fresh fruits, cold meats and rootless vegetables. In this aspect they do not differ from consumers in other countries. The statistical tools applied have proven that respondents, while reporting the dumping of food products mentioned above, also pointed out food spoilage as the most frequent reason for dumping. 

It has been proven that from among five analyzed areas of practices of Polish respondents, food waste due to its spoilage was mainly caused because of inappropriately dealing with food brought home, failure to provide the right food storage conditions or inadequately dealing with uneaten meals. Compliance with the rules of respondents’ personal hygiene as well as hygiene ensured during applied processes of meal preparation there was no influence on food waste due to its spoilage. No statistical dependencies have been proven between the practices consumers presented while shopping i.e., paying attention to the preservation of the cold chain in the case of perishable food products or frozen foods, and dumping food due to its spoilage. 

To minimize the wastage of food in Polish households, greater attention of consumers should be drawn to the conditions of food storage at home. The key factor here is also convincing them to use freezing of uneaten food as an effective method of prolonging the shelf life of food products. Therefore, those two additional aspects should especially be emphasized during educational campaigns aimed at consumers. 

The obtained results as well as the studies of other authors have proven that the problem of food wastage in households is complex and depends on many distinct factors. Thus, to minimize the wastage of food by customers, a wide range of correlative factors should be taken into account.

### Limitations of This Study and Future Research

The advantage of this research is the representative nature of the sample. Therefore, on the basis of the received results, one can draw conclusions about the practices of the adult component of Polish society. However, the study also has some limitations. Firstly, one should remember that respondents did not always report their real reactions to the discussed problem. However, the received results, as proven in the discussion, are, in many aspects, convergent to the results obtained by other researchers. This provides the foundations to qualify the obtained results as reliable. Secondly, while studying the practices of respondents in five selected areas, only some aspects characteristic for a given area were chosen. What should be considerably expanded in future research, is the issue concerning, for example, the conditions of the storage of food products at home or dealing with uneaten meals. Thirdly, as presented in the discussion, the practices of consumers, concerning the frequency of preparing meals at home as well as including some basic food products in their everyday diet, changed noticeably during the pandemic of COVID-19. Thus, the study should be repeated to assess the influence of the COVID-19 pandemic on the present practices of consumers employed while dealing with food, in the context of waste due to its spoilage.

## Figures and Tables

**Table 1:** Socio-demographic characteristics of the respondents.

Feature	Group	Number of Respondents (*n*)	Percentage *(%)*
Gender	women	570	51.1
	men	545	48.9
Age	18–24 y.o.	92	8.3
	25–34 y.o.	212	19.0
	35–44 y.o.	201	18.0
	45–59 y.o.	305	27.4
	60 y.o. and more	305	27.4
Education	elementary	94	8.4
	vocational	356	31.9
	secondary	468	42.0
	higher	197	17.7
Inhabitancy (place of origin)	villages	426	38.2
	Cities up to 50,000	276	24.8
	Cities over 50,000 up to 100,000	82	7.4
	Cities over 100,000 up to 200,000	102	9.1
	Cities over 1200,000 up to 500,000	100	9.0
	Cities over 500,000	129	11,6

**Table 2:** Applied ratings and the ranges of the synthetic indicator relating to respondents’ knowledge and practices and throwing away food frequency.

Rating of Knowledge (K1–13)/Practices (P1–5)	Rating of Throwing away Food Frequency (FTAF_32_)	The Range of Synthetic Indicator (SI)
very good	unsatisfactory	from 0.90 to 1.00
good	satisfactory	from 0.70 to 0.89
satisfactory	good	from 0.50 to 0.69
unsatisfactory	very good	under 0.50

**Table 3:** Average values of the calculated synthetic indicators (SI) for knowledge (K1–13) and the selected areas of practices (P1–P5) of the respondents (*n* = 1115) with assigned ratings.

Area *	Synthetic Indicator (SI)	The Range of SI/Rating of Knowledge and Practices							
		Under 0.5 *Unsatisfactory Grade*		From 0.5 to 0.69 *Satisfactory Grade*		From 0.7 to 0.89 *Good Grade*		From 0.9 to 1.0 *Very Good Grade*	
	Mean (Min./Max.)	*n*	%	*n*	%	*n*	%	*n*	%
K1–13	0.500 (0.189/0.848)	603	54.08	448	40.18	64	5.74	0	0.00
P1	0.500 (0.200/0.960)	579	51.93	263	23.59	248	22.24	25	2.24
P2	0.681 (0.168/0.011)	134	12.02	456	40.90	404	36.23	121	10.85
P3	0.500 (0.194/0.791)	557	49.96	410	36.77	148	13.27	0	0
P4	0.500 (0.001/0.996)	637	57.13	227	20.36	158	14.17	93	8.34
P5	0.500 (0.136/0.914)	583	52.29	390	34.98	139	12.47	3	0.27

* P1—purchases-shop; P2—purchases-home; P3—personal and process hygiene; P4—storage; P5—uneaten meals.

**Table 4:** The mean value of the synthetic indicator (SI) for the frequency of throwing away 32 different food products (FTAF_32_).

Area *	Synthetic Indicator (SI)	The Range of SI/Rating of Frequency of Throwing Away Food							
		Under 0.5 *Very Good Grade*		From 0.5 to 0.69 *Good Grade*		From 0.7 to 0.89 *Satisfactory Grade*		From 0.9 to 1.0 *Unsatisfactory Grade*	
	Mean (Min./Max.)	*n*	%	*n*	%	*n*	%	*n*	%
FTAF_32_	0.500 (0.307/0.951)	628	56.32	316	28.34	150	13.45	21	1.88

* Frequency of Throwing Away Food.

**Table 5:** Regression analysis results showing relations between the frequency of throwing away food and the reported reasons for doing it.

Reasons of Throwing Away Food	% of Indications	Evaluation of Parameters: Frequency of Throwing Away					
		*t*	*p*	β	β SE	−95% CI	+95% CI
thoughtless shopping	15.07	7.734	0.000	0.202	0.026	0.150	0.253
excessive food purchase	17.76			0.190	0.026	0.139	0.241
spoilage of food	51.48	8.333	0.000	0.223	0.027	0.171	0.276
excessive food preparation	21.08	3.268	0.001	0.086	0.026	0.034	0.138
overrun of expiration date	33.36	5.903	0.000	0.156	0.026	0.104	0.207
lack of idea how to use a product	8.07	6.296	0.000	0.164	0.026	0.113	0.216
inappropriate storage	11.49	5.493	0.000	0.143	0.026	0.092	0.194
too large package units	13.72	6.448	0.000	0.169	0.026	0.117	0.220

**Table 6:** Calculated coefficients of the Spearman rank (r) between the frequency of throwing away food and the reasons for doing it.

Reasons for Throwing Away Food	Spearman’s Rank Correlation Coefficient			
	Bread	Fresh Fruit	Smoked Meats	Other Vegetables (Except Root Vegetables)
thoughtless shopping	−0.171 *	−0.183 *	−0.173 *	−0.127 *
excessive food purchase	−0.171 *	−0.185 *	−0.195 *	−0.189 *
spoilage of food	−0.265 *	−0.314 *	−0.257 *	−0.323 *
excessive food preparation	−0.165 *	−0.188 *	−0.130 *	−0.172 *
overrun of expiration date	−0.173 *	−0.202 *	−0.200 *	−0.199 *
lack of idea how to use a product	−0.124 *	−0.073 *	−0.059 *	−0.086 *
inappropriate storage	−0.149 *	−0.105 *	−0.088 *	−0.096 *
too large package units	−0.143 *	0.035	−0.077	−0.081 *

* *p* < 0.05.

**Table 7:** The results of regression analysis showing relations between different areas of practices employed while dealing with food and throwing away food due to its spoilage.

Areas of Practices *	Evaluation of Parameters: Throwing Away Food Due to Its Spoilage					
	*t*	*p*	β	β SE	−95% CI	+95% CI
P1: purchases-shop	−0.877	0.381	−0.026	0.030	−0.084	0.032
P2: purchases-home	4.208	0.000 *	0.135	0.032	0.072	0.198
P3: personal and process hygiene	0.279	0.780	0.009	0.031	−0.052	0.070
P4: storage	2.167	0.030 *	0.066	0.031	0.006	0.126
P5: uneaten meals	2.190	0.029 *	0.066	0.031	0.007	0.130

* P1—purchases-shop; P2—purchases-home; P3—personal and process hygiene; P4—storage; P5—uneaten meals.

**Table 8:** The results of analysis with the use of the logit model showing relations between chosen models of dealing with food and waste due to its spoilage.

Areas of Practices	Evaluation of Parameters: Model Logit					
	LOR **	OR ***	Wald Test	−95% CI	+95% CI	*p*
Free word	−2.0664		29.7804	−2.80860	−1.32426	
P1: purchases-shop	−0.0002	0.9998	0.7567	−0.00075	0.00029	0.384
P2: purchases-home	0.0016 *	1.0016	17.1278	0.00085	0.00239	0.000
P3: personal and process hygiene	0.0001	1.0001	0.0779	−0.00064	0.00085	0.780
P4: storage	1.2355 *	3.4400	4.6762	0.11550	2.35546	0.031
P5: uneaten meals	0.0008 *	1.0008	4.7422	0.00008	0.00161	0.029

* *p* < 0.05; ** LOR-log odds ratio; *** OR-odds ratio.

**Table 9:** The table of the variable “throwing away food due to its spoilage” relevance.

Actual	Predicted		Share of Correctly Predicted Cases
	$\hat{Y}=1$	$\hat{Y}=0$	
Y = 1	377	197	65.679443
Y = 0	262	279	51.571165

## Data Availability

Not applicable.

## Appendix A

**Table A1:** A set of questions taken into account while constructing a synthetic variable concerning the knowledge along with the calculated weights and ranks.

No.	Statement	Answers *	*f* **	Weight of Question	Ranks of the Answers (Min./Max.)
K1	Distribution of temperature inside the refrigerator is even	I strongly agree- **I strongly disagree/I rather disagree**	0.454	0.086	33.5/1053.0
K2	When stored in a refrigerator, fresh meat can be put next to cured meats	I strongly agree- **I strongly disagree/I rather disagree**	0.474	0.083	21.5/1022.0
K3	Storage of raw/unprocessed eggs at room temperature is completely afe	I strongly agree- **I strongly disagree/I rather disagree**	0.414	0.093	28.5/1036.0
K4	Washing of fruits and vegetables reduces the number of microorganisms on their surface	**I strongly agree/I rather agree**- I strongly disagree	0.756	0.039	10.5/914.0
K5	Washings hands with warm water and soap after breaking an egg reduces the risk of food poisoning	**I strongly agree/I rather agree**- I strongly disagree	0.685	0.050	12.5/956.5
K6	Leaving leftovers, e.g., soup or goulash, until it cools down on the countertop for an unlimited period of time poses no danger to health	I strongly agree- **I strongly disagree/I rather disagree**	0.499	0.079	36.0/1013.0
K7	If leftovers look ‘normal’ or smell good, they are still safe and can be eaten	I strongly agree- **I strongly disagree/I rather disagree**	0.203	0.126	50.5/1086.0
K8	The cutting board used for raw meat should be quickly washed with warm water and detergent or put in a dishwasher	**I strongly agree/I rather agree**- I strongly disagree	0.745	0.040	10.0/912.5
K9	In order to get bacteria from hands before touching food, it is enough to wash hands only with cold running water	I strongly agree- **I strongly disagree/I rather disagree**	0.527	0.075	32.0/994.5
K10	Thawed products, e.g., meat, if not used, can be frozen again	I strongly agree- **I strongly disagree/I rather disagree**	0.744	0.040	15.5/836.5
K11	Indication of the correct order of putting purchased products in the cart	**first non-food products, followed by food products that do not require storage in a refrigerator, and finally fresh food that requires storage in low temperatures**	0.512	0.077	272.5/972.5
K12	Indication of the correct storage temperature of fresh poultry meat in the store display window	**from −2 °C to +4 °C**	0.370	0.099	351.5/909.0
K13	Indication of the best place in the refrigerator for storing fresh ground meat	**on the bottom shelf**	0.293	0.112	394.5/952.0

* the correct answers are marked with the bold fonts, ** frequency of correct answers.

**Table A2:** A set of questions taken into account while constructing a synthetic variable concerning the practice along with the calculated weights and ranks.

No.	Question	Answers *	*f* **	Weight of Question	Ranks of The Answers (Min./Max.)
P1a	Frequency of paying attention to the temperature of the refrigerator/refrigerated counter/freezer in the store	**always/usually**-never	0.189	0.316	278.5/1102
P1b	Frequency of putting fresh meat/fish/cured meats in the cart at the end of the shopping	**always/usually**-never	0.344	0.256	184.0/1093.5
P1c	Frequency of use thermal insulation bags when buying frozen foods	**always/usually**-never	0.223	0.303	283.0/1089.0
P1d	Importance of the storage conditions specified by manufacturer in the product label	**definitely relevant/rather relevant**-definitely irrelevant	0.680	0.125	188.75/1054.5
P2a	Frequency of observance of the storage conditions recommended by the manufacturer	**always/usually**-never	0.664	0.358	151.0/1051.5
P2b	Frequency of putting perishable food in a refrigerator immediately after returning from shopping	**always/usually**-never	0.790	0.223	151.0/1051.5
P2c	Frequency of washing of purchased eggs before putting them in the refrigerator	always-**rarely/never**	0.607	0.418	64.5/965.0
P3a	Frequency of use of the same knife (without washing) to cut raw and then cooked meat	always-**rarely/never**	0.556	0.285	50.5/866.5
P3b	Frequency of washing of fruits and vegetables prior to consumption	**always/usually**-never	0.787	0.137	47.0/807.0
P3c	Frequency of washing of hands before meal preparation	**always/usually**-never	0.794	0.133	43.5/785.0
P3d	Method of thawing of products, e.g., meat	**I put it in the fridge and/or in a microwave oven**	0.309	0.445	386.0/943.5
P4	Indication of products that are usually kept in the refrigerator (a multiple-choice question)	Answer cafeteria	**Table A3**	**Table A3**	1.5/1110.5
P5a	Uneaten meals: leaving in a pot on the stove/or in the oven until they are eaten	always-**rarely/never**	0.542	0.231	10.0/976.0
P5b	Uneaten meals: putting meals that are still warm in the refrigerator	always-**rarely/never**	0.821	0.090	4.0/763.0
P5c	Uneaten meals: cooling down to room temperature and then putting them in the fridge	**always/usually**-never	0.494	0.255	53.0/1013
P5d	Uneaten meals: freezing	**always/usually**-never	0.158	0.424	127.0/1102.0

* the correct answers are marked with the bold fonts, ** frequency of correct answers.

**Table A3:** Frequency of showing correct answers (*f*) regarding the storage of food products (P:4).

Food Product *	*f* **	Weight	Food Product *	*f* **	Weight
UHT milk	0.415	0.141	bread	0.963	0.009
**lettuce**	0.481	0.126	**cured meats**	0.915	0.021
potatoes	0.974	0.006	**fresh milk**	0.810	0.046
**fresh poultry**	0.815	0.045	carrots	0.645	0.065
garlic	0.866	0.032	**butter**	0.840	0.039
bananas	0.890	0.027	tomatoes	0.645	0.086
peppers	0.692	0.074	**eggs**	0.785	0.052
fresh herbs	0.904	0.023	onion	0.833	0.040
**fresh fish**	0.766	0.057	**opened juice containers**	0.633	0.089
**yoghurt, buttermilk, etc.**	0.910	0.022			

* the food products that should be kept at refrigerator temperature are marked with the bold fonts;,** frequency of correct answers.
